# Seroprevalence and Risk Factors for Cysticercosis in Mexican Americans in Starr County, Texas

**DOI:** 10.3390/pathogens13110988

**Published:** 2024-11-12

**Authors:** Megan M. Duffey, Elise M. O’Connell, Morgan Jibowu, Fanny E. Moron, Lauren M. Leining, Nina L. Tang, Craig L. Hanis, Eric L. Brown, Sarah M. Gunter

**Affiliations:** 1 Department of Medicine, Section of Infectious Diseases, Baylor College of Medicine, Houston, TX 77030, USA; 2 National School of Tropical Medicine, Section of Tropical Medicine, Department of Pediatrics, Baylor College of Medicine, Houston, TX 77030, USA; morgan.jibowu@bcm.edu (M.J.); lauren.m.leining@uth.tmc.edu (L.M.L.); sarah.gunter@bcm.edu (S.M.G.); 3 William T. Shearer Center for Human Immunobiology, Texas Children’s Hospital, Houston, TX 77030, USA; 4 Laboratory of Parasitic Diseases, National Institute of Allergy and Infectious Diseases, National Institutes of Health, Bethesda, MD 20892, USAnina.tang@bcm.edu (N.L.T.); 5 Center for Infectious Disease, Department of Epidemiology, University of Texas School of Public Health, Houston, TX 77030, USA; eric.l.brown@uth.tmc.edu; 6 Department of Radiology, Baylor College of Medicine, Houston, TX 77030, USA; fmoron@bcm.edu; 7 Human Genetics Center, Department of Epidemiology, University of Texas School of Public Health, Houston, TX 77030, USA; craig.l.hanis@uth.tmc.edu

**Keywords:** taeniasis, tropical medicine, cysticercosis, neurocysticercosis, cestode infections

## Abstract

Cysticercosis is a parasitic infection and neglected tropical disease caused by *Taenia solium*, or the pork tapeworm. Cysticercosis with central nervous system involvement, or neurocysticercosis, is a leading cause of chronic headaches and epilepsy in endemic regions, including Latin America and Asia. In the United States, the epidemiology of cysticercosis has not been well described. We conducted a cross-section serosurvey of Mexican-American adults residing along the Texas–Mexico border (Starr County, Texas) and identified an overall seroprevalence of 7.4% (45/605) for cysticercosis. Brain imaging studies conducted on seropositive study participants identified lesions consistent with calcified neurocysticercosis in 2 of the 45 seropositive individuals. Female sex (*p* = 0.021), employment in healthcare, caregiving, or social service (*p* = 0.002), and indoor occupation (*p* < 0.001) were found to be significantly associated with seropositivity. Further study is needed to evaluate the burden of neurocysticercosis and local transmission risk in this community.

## 1. Introduction

Cysticercosis is a neglected tropical disease (NTD) caused by pork tapeworms (*Taenia solium*). Humans become infected by the ingestion of eggs shed in the stool of a person harboring an adult pork tapeworm infection in their small intestine. Following exposure, metacestodes (colloquially known as cysts) can develop in tissues and organs throughout the body. When cysts develop in the central nervous system, the infection is called neurocysticercosis (NCC). Clinical presentation of NCC varies largely based on the number and location of cysts and can range from asymptomatic to significant neurologic sequelae. Symptoms can include headaches, increased intracranial pressure, and/or seizures/epilepsy [1]. Parenchymal NCC typically presents as seizures, while extraparenchymal NCC can present as headache, dizziness, or increased intracranial pressure from hydrocephalus [1]. The identification of extraparenchymal infection and appropriate treatment is critical, as mortality in these cases is 16% [2]. Chronic symptoms caused by this infection have been associated with significant socioeconomic loss in heavily affected areas [3]. Cysticercosis involving the subcutaneous tissue and muscle is often asymptomatic.

Cysticercosis and NCC are endemic in low- and middle-income countries in Latin America, sub-Saharan Africa, and South, East, and Southeast Asia [1]. In the United States, the majority of recognized NCC cases have been described in immigrants and travelers who acquired the infection in endemic countries [4]. While sporadic reports of autochthonous infection have been documented in the USA [5], the epidemiology of cysticercosis and autochthonous transmission dynamics remains ill defined.

The Texas–Mexico border has been identified as a high-risk area for autochthonous NTD transmission, including cysticercosis and NCC [6]. Starr County, Texas, located on the Texas–Mexico border, consists almost entirely of Mexican Americans, has a high poverty rate (34.9%), and is designated as a Health Professional Shortage Area (HPSA) [7,8]. Other NTDs, such as Chagas disease, have been described in this population [9]. These factors, combined with a high rate of immigration from endemic regions, may put this population disproportionately at risk for cysticercosis and NCC. In the following study, we investigated the prevalence of cysticercosis and NCC in a population living along the Texas–Mexico border. Our overarching study goal was to better define the epidemiology of this disease in an at-risk population living in the USA.

## 2. Materials and Methods

### 2.1. Serological Testing and Neuroimaging

We conducted a cross-sectional serologic survey of Mexican-American adults residing in Starr County, Texas, who were enrolled between 2018 and 2020. This convenience sampling utilized samples from an existing cohort that was originally established to investigate the relationship between the gut microbiome and diabetes status [10]. From this cohort (*n* = 616), plasma from 605 participants (98.2%) was available for cysticercosis serology testing. Samples were tested for reactivity against cysticercosis-specific antigens T24H, GP50, and Ts18var3 via a triplex enzyme-linked immunoassay (ELISA). This triplex ELISA was previously validated and found to have 98% positive and 100% negative concordance when compared to an electroimmunotransfer blot (EITB) as the reference standard [11]. Brain magnetic resonance imaging (MRI) with and without intravenous contrast was performed in qualifying seropositive participants. The brain MRI protocol included axial T1- and T2-weighted images, axial FLAIR (fluid attenuation inversion recovery), and SWI (susceptibility weighted imaging) to evaluate for calcifications and T2-FIESTA (fast imaging employing steady-state acquisition) to assess for subarachnoid NCC. The brain MRI was independently read by two neuroradiologists, one with more than 20 years of experience (F.M.). This study was reviewed and approved by the institutional review board (IRB) at the University of Texas Health Science Center (HSC-SPH-06-0225).

### 2.2. Risk Factor Analysis

To better understand community- and individual-level drivers for seropositivity, we conducted a risk factor analysis using self-reported survey data and neighborhood-level variables. The survey data included information on participant demographic, socioeconomic, and lifestyle variables. Each participant’s address at enrollment was geocoded to assess for community-level variables, including the area deprivation index (ADI) at a census tract level and if their residence was in a known colonia, a community with substandard housing and a lack of basic municipal services commonly found on the Texas–Mexico border [12]. ADI is a composite score (ranging from 1 to 10) used to assess neighborhood socioeconomic disadvantage [13]. We evaluated the association between these variables and cysticercosis seropositivity using Chi-square and Kruskal–Wallis tests for categorical values and the Mann–Whitney test for continuous variables. All statistical analyses were conducted in Stata v16 [Stata Corp, College Station, TX, USA].

## 3. Results

We identified an overall seropositivity of 7.4% (45/605) for cysticercosis in the cohort. Of the 45 participants testing positive, 40 were positive for Ts18var3 only, 1 was positive for T24H only, 1 was positive for Ts18var3 and T24H, and 3 were positive for all three antigens. Female sex and type of occupation were found to be significantly associated with positive cysticercosis serology (Table 1). Of the 45 seropositive participants, 39 (86.7%) were female (*p* = 0.021), though it should be noted that the overall cohort is 71.7% female. Additionally, 26 (58%) were employed in healthcare, caregiving, or social service (*p* = 0.002), and 42 were employed in predominately indoor occupations (*p* < 0.001).

**Figure 1:**
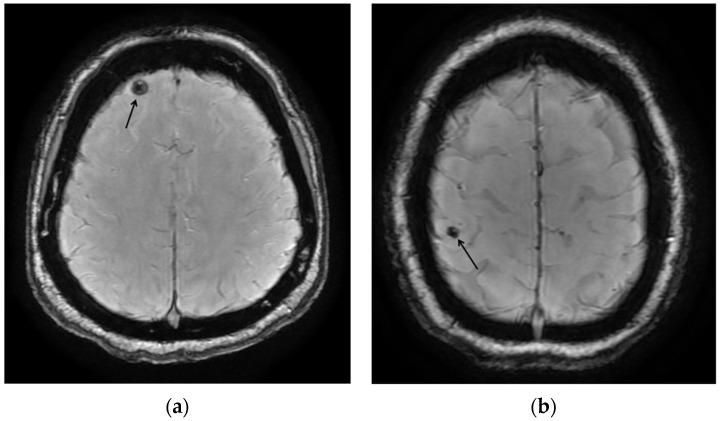
MRI images: (**a**) the susceptibility-weighted image shows a round hypointense calcification (arrow) in the right anterior frontal lobe, with no surrounding edema or mass effect, consistent with the calcified nodular stage of NCC; (**b**) the susceptibility-weighted image shows a round hypointense calcification (arrow) in the right superior parietal lobule, with no surrounding edema or mass effect, consistent with the calcified nodular stage of NCC.

A total of 37 participants underwent MRI (82% of seropositive participants), 4 participants declined the MRI, and 4 were lost to follow-up. We identified two participants with calcifications consistent with inactive NCC (5.4%). One participant exhibited focal calcification in the right superior parietal lobe, while the other participant had a calcification in the right frontal lobe (Figure 1). Both participants were born in Mexico, were employed in healthcare, caregiving, or social service, and have lived in Starr County for more than 30 years. In addition, both participants were positive for Ts18var3 but not T24H or GP50 (Table 2).

## 4. Discussion

We found an overall cysticercosis seroprevalence of 7.4%, with two cases of confirmed calcified NCC based on neuroimaging. The seropositivity of a population likely reflects a combination of parasite exposure, muscle or subcutaneous disease, old calcified NCC, and least common viable or degenerating NCC. Additionally, false-positive serologic results are possible. The seroprevalence identified in this cohort is similar to rates described in endemic countries in Central and South America. Previous studies have found a cysticercosis seroprevalence of 4.9–12.2% in Mexico and 5–35% in Peru [14,15]. Studies of cysticercosis prevalence in the United States are limited to specific populations and are largely not generalizable. Previous work has identified a seroprevalence of 1.8% in migrant farmers and local residents in rural southern California [16], 10% in Hispanic and Haitian migrant farmworkers in North Carolina [17], and 1.3% in an Orthodox Jewish community [18].

In our study, two seropositive participants had MRI findings consistent with calcified NCC disease; both were reactive to only the Ts18var3 antigen. Previous research has identified an association with Ts18var3 reactivity and parenchymal disease, consistent with our findings [11]. Notably, neuroimaging cannot detect already-healed lesions or lesions that may be present elsewhere in the body, such as in subcutaneous or muscle cysticercosis. Previous studies have demonstrated that a relatively small portion of seropositive individuals have evidence of infection detected with neuroimaging [19,20,21], and our findings are consistent with these.

Seroprevalence studies, such as this one, can provide useful information on the burden and distribution of *T. solium* exposure in a community, especially when distinct risk factors are identified [15]. In our cohort, we found that female sex, indoor occupations, and occupations specifically in the healthcare, caregiving, and social service domains were significantly associated with cysticercosis seropositivity. Previous research into the association between cysticercosis and sex is mixed, with studies identifying no association or positive association in either men or women [19,21,22,23,24]. The associations found with occupations in healthcare, caregiving, and social service domains could possibly represent a risk for transmission through common tasks such as aiding in toileting and bathing, as cysticercosis is typically acquired through fecal–oral contamination by someone with intestinal *T. solium* tapeworm infection.

Interestingly, we did not identify living in high-poverty communities (ADI or colonias) as a significant risk factor for seropositivity, perhaps due to the general high poverty rate in the area impacting our ability to detect significant differences between groups (mean ADI for seropositive, 9.33, and seronegative, 9.26). Household income was also found to not be significant between the seropositive and seronegative participants. However, specific risk factors such as travel to endemic regions and/or activities with non-commercial pork have not yet been assessed in this population.

Our study has some noteworthy limitations. The convenience sampling study design may have resulted in a cohort not representative of the population of Starr County or the surrounding communities. Further research should be conducted to determine if our findings are consistent with the larger Texas–Mexico border community. Additionally, due to the nature of serologic testing, we cannot determine if seropositive participants with no neuroimaging findings had this result due to a false positive, muscle or subcutaneous disease, or a previous lesion that has since healed.

Given our preliminary findings and the limitations of this testing, there is a critical need to further investigate the burden of disease in this community and the risk of local transmission along the Texas–Mexico border. Determining whether there is autochthonous transmission in these communities is an important next step in defining the local risk of disease. The answers to these questions can be used to inform public health interventions to mitigating disease transmission and increase targeted screening in high-risk populations to prevent the morbidity and mortality of this often-hidden disease.

## Figures and Tables

**Table 1:** Demographic and risk factor analysis of cysticercosis seropositivity. Values are no. (%) except as indicated.

Demographic		Cysticercosis Seronegative	Cysticercosis Seropositive	*p*-Value
		*n* = 560	*n* = 45	
Sex				0.021 ^⍭^
	Male	165 (29.5%)	6 (13.3%)	
	Female	395 (70.5%)	39 (86.7%)	
Age (years)				0.424 *
	30–39	68 (12.1%)	3 (6.7%)	
	40–49	201 (35.9%)	16 (35.5%)	
	50–59	207 (37.0%)	19 (42.2%)	
	>60	84 (15.0%)	7 (15.6%)	
Years of education				0.954 ^⍭^
	≤12	446 (79.6%)	36 (80.0%)	
	>12	114 (20.4%)	9 (20.0%)	
Income (household)				0.284 *
	<USD 20,000	223 (39.8%)	21 (46.7%)	
	USD 20,001–USD 30,000	129 (23.0%)	10 (22.2%)	
	USD 30,001–USD 40,000	50 (8.9%)	6 (13.3%)	
	USD 40,001–USD 50,000	54 (9.6%)	4 (8.9%)	
	>USD 50,001	95 (17.0%)	4 (8.9%)	
Health insurance status				0.217 ^⍭^
	Uninsured	297 (53.0%)	20 (44.4%)	
	Insured	253 (45.2%)	25 (55.6%)	
Employment status				0.352 *
	Working full-time	246 (44.9%)	22 (48.9%)	
	Working part-time	108 (19.3%)	13 (28.9%)	
	Unemployed	140 (25.0%)	7 (15.6%)	
	Retired	20 (3.6%)	1 (2.2%)	
	Extended sick leave	1 (0.2%)	0 (0%)	
	Disabled	36 (6.4%)	2 (4.4%)	
Current or previous occupation				0.002 *
	Management, business, creators, teaching, administration	71 (12.7%)	6 (13.3%)	
	Healthcare, caregiving, social service	189 (33.8%)	26 (57.8%)	
	Sales, customer service	62 (11.1%)	8 (17.8%)	
	Maintenance, construction, farming, transportation	165 (29.5%)	3 (6.7%)	
	Other (self-employed, housewife, or missing data)	73 (13.0%)	2 (4.4%)	
Outdoor occupation				<0.001 ^ς^
	No	394 (70.4%)	42 (93.3%)	
	Yes	166 (29.6%)	3 (6.7%)	
Residency in colonia				0.904 ^⍭^
	No	281 (50.2%)	23 (51.1%)	
	Yes	279 (49.8%)	22 (48.9%)	
ADI (area deprivation index) Mean (95%CI)		9.26 (9.20–9.33)	9.33 (9.12–9.54)	0.734 ^#^
Years lived in Starr County				0.957 *
	3–24	150 (26.8%)	7 (15.6%)	
	25–32	134 (23.9%)	21 (46.7%)	
	33–41	145 (25.9%)	6 (13.3%)	
	42–65	131 (23.4%)	11 (24.4%)	
Marriage status				0.585 ^⍭^
	Not married	165 (29.5%)	15 (33.3%)	
	Married	395 (70.5%)	30 (66.7%)	
Birthplace				0.376 ^⍭^
	Mexico	396 (70.7%)	29 (64.4%)	
	USA	164 (29.3%)	16 (35.6%)	

^⍭^ indicates χ^2^ test; ^ς^ indicates Fisher’s exact test; * indicates Kruskal–Wallis test; and ^#^ indicates Mann–Whitney test.

**Table 2:** Demographics, risk factors, serological testing results, and neuroimaging results.

Study ID	Sex	Income >USD 30,000	Birthplace	Age	Current or Previous Occupation Category	Outdoor Occupation	Ts18var3	T24H	GP50	MRI Results
NC0120	Female	No	Mexico	40–59	Sales, customer service, and community-oriented	No	Yes	No	No	Negative
NC0067	Female	No	Mexico	40–59	Healthcare, caregiving, and social service	No	Yes	Yes	No	Negative
NC0260	Male	Yes	United States	40–59	Healthcare, caregiving, and social service	No	Yes	No	No	Negative
NC0061	Female	No	Mexico	40–59	Healthcare, caregiving, and social service	No	Yes	No	No	Negative
NC0354	Female	No	United States	40–59	Healthcare, caregiving, and social service	No	Yes	No	No	Negative
NC0127	Female	No	United States	40–59	Supervisors, creators, teaching, professionals, and office	No	Yes	No	No	Negative *
NC0108	Female	No	Mexico	40–59	Healthcare, caregiving, and social service	No	Yes	No	No	Negative *
NC0075	Female	No	United States	40–59	Healthcare, caregiving, and social service	No	Yes	No	No	Negative
NC0172	Male	Yes	United States	40–59	Supervisors, creators, teaching, professionals, and office	No	Yes	No	No	Negative
NC0095	Female	No	Mexico	40–59	Healthcare, caregiving, and social service	No	Yes	No	No	Negative
NC0074	Female	Yes	Mexico	40–49	Healthcare, caregiving, and social service	No	Yes	No	No	Positive
NC0098	Female	Yes	Mexico	40–59	Sales, customer service, and community-oriented	No	Yes	Yes	No	Negative
NC0126	Female	No	Mexico	40–59	Healthcare, caregiving, and social service	No	Yes	No	No	Positive
NC0363	Female	No	United States	>60	Healthcare, caregiving, and social service	No	Yes	Yes	Yes	Negative
NC0081	Female	No	Mexico	40–59	Healthcare, caregiving, and social service	No	Yes	Yes	Yes	Negative
NC0179	Female	No	Mexico	>60	Outdoor and manual labor, and workforce	Yes	Yes	No	No	Negative
NC0096	Female	Yes	United States	40–59	Healthcare, caregiving, and social service	No	Yes	No	No	Negative
NC0340	Female	Yes	Mexico	40–59	Healthcare, caregiving, and social service	No	Yes	No	No	Negative
NC0090	Female	No	Mexico	40–49	Sales, customer service, and community-oriented	No	Yes	No	No	Negative
NC0289	Male	No	Mexico	40–59	Sales, customer service, and community-oriented	No	Yes	No	No	Negative *
NC0089	Female	Yes	United States	<40	Supervisors, creators, teaching, professionals, office	No	Yes	No	No	Negative
NC0259	Male	No	United States	60–69	Outdoor and manual labor, and workforce	Yes	Yes	No	No	Negative
NC0228	Male	Yes	Mexico	40–59	Outdoor and manual labor, and workforce	Yes	Yes	No	No	Negative
NC0119	Female	No	Mexico	40–59	Sales, customer service, and community-oriented	No	Yes	No	No	Negative
NC0129	Female	No	United States	40–59	Healthcare, caregiving, and social service	No	Yes	No	No	Negative
NC0160	Female	Yes	Mexico	40–59	Healthcare, caregiving, and social service	No	Yes	No	No	Negative
NC0123	Female	Yes	Mexico	40–59	Sales, customer service, and community-oriented	No	Yes	No	No	Negative
NC0485	Female	Yes	United States	40–59	Healthcare, caregiving, and social service	No	Yes	No	No	Negative
NC0161	Female	No	Mexico	40–59	Healthcare, caregiving, and social service	No	Yes	No	No	Negative
NC0454	Female	No	Mexico	>60	Healthcare, caregiving, and social service	No	Yes	Yes	Yes	Negative
NC0262	Female	No	Mexico	40–59	Supervisors, creators, teaching, professionals, and office	No	Yes	No	No	Negative
NC0117	Female	No	Mexico	40–59	Healthcare, caregiving, and social service	No	Yes	No	No	Negative
NC0464	Female	No	Mexico	40–59	Healthcare, caregiving, and social service	No	Yes	No	No	Negative *
NC0300	Male	No	Mexico	40–59	Sales, customer service, and community-oriented	No	Yes	No	No	Negative
NC0072	Female	No	Mexico	<40	Healthcare, caregiving, and social service	No	Yes	No	No	Negative
NC0097	Female	No	Mexico	40–59	Healthcare, caregiving, and social service	No	Yes	No	No	Negative
NC0164	Female	Yes	United States	<40	Supervisors, creators, teaching, professionals, and office	No	Yes	No	No	Negative

Note: Age is at time of enrollment in parent study; * MRI completed without contrast.

## Data Availability

The data that support the findings are available on request from the corresponding author, M.M.D. The data are not publicly available due to them containing protected health information (PHI).
